# Factors associated with non‐participation in the Healthy Cognitive Ageing Project

**DOI:** 10.1002/alz.70169

**Published:** 2025-04-28

**Authors:** Sarah Assaad, Shabina Hayat, Carol Brayne, Paola Zaninotto, Andrew Steptoe

**Affiliations:** ^1^ Department of Epidemiology and Public Health, Institute of Epidemiology and Health Care University College London London UK; ^2^ Department of Behavioural Science and Health, Institute of Epidemiology and Health Care University College London London UK; ^3^ Department of Psychiatry University of Cambridge Cambridge UK

**Keywords:** aging, cognition, cohort, dementia, non‐participation, non‐response, study weights

## Abstract

**BACKGROUND:**

Understanding cognitive decline trajectories is crucial for dementia prevention, as many cases go undetected. Identifying participation biases in such studies is essential for data validity.

**METHODS:**

We examined non‐participation correlates in the Healthy Cognitive Ageing Project (HCAP), a sub‐study of the English Longitudinal Study of Ageing (ELSA). We compared sociodemographic and health characteristics of invited, interviewed, and non‐interviewed individuals, and assessed the impact of sample weights.

**RESULTS:**

Of 1778 ELSA members invited in 2018, 1273 (72%) participated. Participants were similar to the invited sample in sociodemographics but were younger, had fewer daily living difficulties, and had better cognition. Non‐participation was linked to difficulties in daily living (odds ratio 1.78), dementia (1.55), and psychiatric conditions (1.34). Weighted analyses highlighted differences in disability and cognition.

**DISCUSSION:**

Non‐participation in cognitive studies is not random, lowering response and retention rates, and requiring adjustments to data analysis beyond the use of weights.

**Highlights:**

- We compared the sociodemographics of invited, interviewed, and non‐interviewed individuals.
- We used sample weights to assess differences in participants' characteristics.
- We found non‐participation linked to daily living difficulties, dementia, and psychiatric conditions.

## 1 BACKGROUND

With the global increase in life expectancy and the proportion of older people within the population, the number of people experiencing dementia is on the rise, given its close relationship to age.^1^ It is estimated that around 57 million people in the world will be living with dementia in 2024. This is projected to nearly triple by 2050, reaching around 153 million. The most robust evidence on the risk and protective factors for dementia comes from large‐scale longitudinal observational studies from across the world.^2^ This is because even in high‐income countries, a significant proportion of those living with dementia are not known to clinical services.^3^, ^4^, ^5^ Estimates based on clinical observation are therefore unreliable. The quality of longitudinal population studies varies and can be difficult to assess. Study samples need to be representative of the population at large, including those in care settings, to allow for robust prevalence and incidence estimates.

Pertinent issues related to longitudinal population‐based studies in older populations are the potential bias of initial non‐response, known as selection and/or participation biases,^6^ and the non‐random attrition from the survey over time (i.e., loss‐to‐follow‐up or dropout).^7^ Participation bias can compromise both the internal and external validity of a study,^8^ and both non‐response and attrition can underestimate the prevalence of cognitive impairment and dementia in the sample.^9^ Although challenging to eliminate unless response rates at the outset are very high and attrition is limited, the risk of biases can be reduced through study design and statistical techniques including informed sensitivity analyses. While research has shown that a range of factors, including cognitive impairment and dementia, can be linked to attrition^10^, little evidence is available on factors associated with initial non‐response in aging cohort studies. In this paper, we focus on the latter.

**RESEARCH IN CONTEXT**

1. **Systematic review**: The authors conducted a comprehensive literature review using traditional sources such as PubMed, focusing on studies related to cognitive decline and participation biases in aging populations. Relevant studies on sociodemographic and health characteristics influencing study participation were identified and cited.
2. **Interpretation**: Our findings highlight that non‐participation in cognitive studies among older adults is not random and is influenced by factors such as daily living difficulties, dementia, and psychiatric conditions. This underscores the importance of considering these biases in study design and data interpretation to ensure valid and generalizable results.
3. **Future directions**: The study suggests several avenues for future research, including: (a) developing strategies to enhance participation among underrepresented groups; (b) exploring the impact of non‐participation on study outcomes; and (c) refining weighting and imputation methods to better represent diverse populations in cognitive aging research.

The Harmonized Cognitive Assessment Protocol (HCAP) network is an international research collaboration aimed at better measurement and identification of cognitive impairment and dementia in the general population.^11^ HCAP involves the administration of a detailed battery of standardized cognitive and neuropsychological assessments in representative population‐based samples of older adults around the world.^11^, ^12^, ^13^, ^14^ This approach facilitates harmonized investigations of cross‐country variation in risk and protective factors of cognitive function in later life and dementia.

A sub‐sample of participants in the English Longitudinal Study of Ageing (ELSA) aged 65 and older in 2018 were invited to complete the HCAP battery.^12^ Individuals in the ELSA‐HCAP study were selected using a systematic sampling protocol, and response rates were high, but 28% of people invited did not take part. To permit generalization to the full ELSA sample, it is important to characterize those who did and did not take part in the study. In the cohort profile paper of the ELSA‐HCAP study,^12^ details on the study rationale, participant selection, and cognitive measures were presented, including a gender‐specific analysis of the sociodemographic, lifestyle, and health characteristics of the ELSA‐HCAP participants and gender‐based weighted mean and standard deviations of the cognitive tests.

Here, we present a detailed analysis of baseline characteristics by interview status (invited, interviewed, and not interviewed) and investigate the factors associated with non‐response or participation bias. We also compared the participants’ characteristics using weighted and unweighted data to determine the effect of weights on the sample description. This work will inform future analysis using the ELSA‐HCAP data for both national and cross‐country analyses.

## 2 METHODS

### 2.1 Study design

ELSA is a large, multidisciplinary study of people aged 50 and over living in private households initiated in 2002 aiming for national representation excluding care settings. The original ELSA sample was drawn from participants in the Health Survey for England (HSE) between 1998 and 2001.^15^ Participants are typically interviewed every 2 years in their own homes (or care settings should they move during follow‐up) using a computer assisted personal interview (CAPI). Detailed descriptions of the recruitment and methods have been presented elsewhere.^15^

The ELSA Healthy Cognitive Ageing Project (ELSA‐HCAP) (https://www.elsa‐project.ac.uk/hcap) is a sub‐study of ELSA and part of an international HCAP program (https://hcap.isr.umich.edu/) aiming to map cognition and cognitive change and was first implemented in 2018 (between 15 January and 8 April).^12^, ^16^ In this study, we ensured a representative sample by inviting participants from diverse sociodemographic backgrounds. Efforts were made to minimize barriers to participation, such as providing accessible interview formats and considering health and mobility challenges and time/availability for the interview. Additionally, a designated individual (usually a family member) was approached when eligible participants did not have the capacity to consent and were asked to provide agreement for participation on their behalf. In the execution phase, we employed inclusive practices to engage groups from different sociodemographic and economic backgrounds. During data preparation, we included weights to account for potential biases and ensure that findings accurately reflect the diverse population when data are analyzed and interpreted.

### 2.2 Sample derivation

**FIGURE 1:**
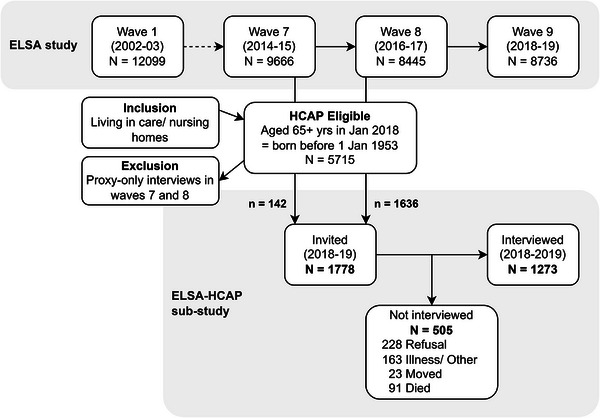
The ELSA‐HCAP sample derivation from ELSA waves 7 and 8. This figure shows the waves in ELSA (1, 7, 8, and 9) with details of study dates and participant numbers. It also shows the derivation of the HCAP sample from ELSA waves 7 and 8 with details of study dates, eligible participants, and inclusion and exclusion criteria. Finally, the figure shows the total invited, interviewed, and not interviewed participants (with reasons for refusal). ELSA, English Longitudinal Study of Ageing; HCAP, Healthy Cognitive Ageing Project.

The rationale and methodology of ELSA‐HCAP have been published elsewhere.^12^ Briefly, individuals were eligible if they were ELSA core members aged 65 and older in January 2018 (start of fieldwork for ELSA‐HCAP) (Figure 1). This included people living in care or nursing homes provided they had lived in the community when first recruited to ELSA, and had the capacity to consent, or if a consultee/family member confirmed that the participant would want to take part in the study. Additionally, all those invited had completed a respondent interview (not informant‐only interview) at wave 8 (2016–2017) of the ELSA study (*n* = 1636) or, alternatively, at wave 7 (2014–2015) (*n* = 142) for those who did not have an interview at wave 8 due to reasons like having an illness/having moved. This is to ensure that participants were able to undertake the cognitive assessment in the ELSA‐HCAP study. Oversampling was done from those who had dementia or low cognitive function based on the cognitive information in the ELSA study.^12^ The final invited sample consisted of 1778 participants, of whom 1273 were interviewed in ELSA‐HCAP (Figure 1). The study response rate was 75.6% among those who were invited and reachable (*N* = 1684), with variations in rates based on cognition at recruitment measured using the 27‐item modified Telephone Interview Cognitive Status (mTICS27) score, with a higher score reflecting better cognition (Table 1).

**TABLE 1:** Response rate in ELSA‐HCAP by cognitive level at recruitment (mTICS27 score) assessed in ELSA waves 7 and 8.

	Invited	Reachable^a^	Interviewed	Response rate (in invited)	Response rate (in reachable^a^)
Cognition at recruitment	*n*	*n*	*n*	%	%
Low (mTICS27 0–6)	277	235	118	42.6	50.2
Moderate (mTICS27 7–11)	491	462	353	71.9	76.4
Normal (mTICS27 12–27)	1010	987	802	79.4	81.3
Total	1778	1684	1273	71.6	75.6

Abbreviation: ELSA, English Longitudinal Study of Ageing; HCAP, Healthy Cognitive Ageing Project; mTICS27, 27‐item modified Telephone Interview Cognitive Status.

^a^ Excluding 91 deaths and three cases moved outside Britain out of the invited sample.

### 2.3 Study variables

#### 2.3.1 Sociodemographic and health characteristics

The sociodemographic characteristics of the invited sample (ELSA wave 7 or 8) included age (in years), gender (men vs. women), marital status (married/with partner vs. not married; the latter group includes widowed, divorced/separated, single/never married), ethnicity (White vs. non‐White), education (some vs. no qualifications), total net income (in GBP per month), and working status (yes vs. no). Measures of health behaviors included cigarette smoking (past/current vs. never), alcohol consumption (number of days per week, dichotomized into < 5 and 5+ days/week), and engagement in moderate or vigorous physical activities categorized into two groups (hardly ever/never vs. regularly/frequently).

Disability measures consisted of eyesight, hearing, and mobility problems (yes vs. no), and having at least one difficulty in basic/instrumental activities of daily living (ADL/IADL). The ADL include six measures of difficulties in eating, bathing, dressing, using the toilet, getting in and out of bed, and walking across a room. The IADL includes eight measures of difficulties in preparing a meal, shopping, making telephone calls, taking medication, doing work around the house and garden, managing finances, communication, and recognizing when in physical danger.

Other physical health variables included self‐rated physical health (fair/poor vs. good/excellent), limiting longstanding illness (yes vs. no), physician‐diagnosed conditions (such as diabetes, hypertension, cardiovascular disease [CVD], and dementia/Alzheimer's disease [AD]). Additionally, mental well‐being was evaluated with depressive symptoms, assessed using the eight‐item version of the Centre for Epidemiological Studies Depression Scale (CES‐D8) with a 4+ cutoff indicating a positive case.^15^

#### 2.3.2 Cognition at recruitment

In addition to a self‐rated assessment of memory (dichotomized into fair/poor vs. good/excellent), participants completed a set of cognitive tests at recruitment (ELSA wave 7 or 8) to measure memory (orientation in time and immediate and delayed word‐list recall tests), fluid intelligence (number series test), and executive function (backward counting, animal naming, serial 7s subtractions, and object naming tests). Details on these tests are provided in Table S1. To assess overall cognition, the mTICS27 score was computed based on the method implemented within the Health and Retirement Study (HRS),^17^, ^18^ and the details are provided in Supporting Information 1.

### 2.4 Statistical analysis

Using complete cases and unweighted data, first, we compared the profiles of the invited and interviewed samples, using z‐tests for proportions and means to detect significant differences in group‐based comparisons of percentages and means for each categorical and continuous variable, respectively. A supplementary analysis was conducted comparing the profiles of the interviewed sample to the eligible participants (*N* = 5715) in ELSA waves 7 and 8 (Figure 1). Second, we compared participants to non‐participants using chi‐squared tests and independent samples *t*‐test for each categorical and continuous variable, respectively. Factors associated with non‐participation at the bivariate level with *p*‐value ≤ 0.2 were eligible for multivariable analysis, including age and gender as universal confounders.

Binary logistic regression models were used to determine the association of each factor with non‐participation in the invited sample, adjusting for age, gender, education, marital status, and income. A sensitivity analysis was conducted, repeating the models in the invited sample but excluding those who had died (*n* = 91) or moved out of Britain (*n* = 3). Results are presented in terms of odds ratios (ORs) and 95% confidence intervals (CIs).

Finally, we compared the baseline characteristics of the ELSA‐HCAP participants using weighted and unweighted data. Sample weights are available in the ELSA‐HCAP dataset and were originally derived by combining design weights and non‐response weights and were subject to a calibration procedure for further adjustments. Non‐response weights were derived from a model including variables such as cognition, age, region, ethnicity, working status, long‐standing illness, self‐reported health, hearing and memory measures, and physical activity.^16^ The use of weights in descriptive and inferential data analyses help preserve the representation of the HCAP sample toward the 65+ older adult population in ELSA, and consequently, facilitate cross‐country comparisons with similar population definition. More details on the weights’ derivation are available in the Cohort profile paper^12^ and the technical report.^16^

All analyses were performed using STATA version 17, and the significance level was set at an alpha of 0.05.[Table alz70169-tbl-0002]

## 3 RESULTS

### 3.1 Characteristics of invited versus interviewed samples

The cognitive profile was significantly different between the two groups, where interviewed participants performed on average better than the invited sample on the cognitive tests, except for the number series test measuring attention and numeric reasoning (Table 2). Furthermore, participants who were interviewed were, on average, slightly younger than the invited sample (74.7 vs. 75.6 years, *p* < 0.01), and a lower proportion of participants reported one or more ADL and IADL difficulties compared to those invited (22% and 25% vs. 25% and 30%, respectively *p *< 0.01). However, participants did not differ from the invited sample with respect to other sociodemographic characteristics, health behaviors, disability, and physical health. A similar comparison of baseline characteristics between the eligible and interviewed samples was conducted (Table S2), the results of which are discussed in Supporting Information 2.

**TABLE 2 alz70169-tbl-0002:** Differences in profiles between invited, interviewed, and not interviewed samples in ELSA‐HCAP.

Characteristics Total *N*	Invited (1) 1778	Interviewed (2) 1273	Not interviewed (3) 505	*p*‐Value (1) vs. (2)	*p*‐Value (2) vs. (3)
**Sociodemographics**					
Age (in years), *mean* (*SD*)	*75.6* (*8.3*)	*74.7* (*7.5*)	*77.8* (*9.7*)	*	*
Gender (women)	990 (55.7)	700 (55.0)	290 (57.4)	0.58	0.35
Marital status (not married)	686 (38.6)	463 (36.4)	223 (44.2)	0.09	*
Ethnicity (White)	1719 (96.7)	1231 (96.7)	488 (96.6)	1.00	0.94
Education (no qualifications)	639 (36.4)	416 (33.1)	223 (44.8)	0.09	*
Working status (not in work)	1598 (89.9)	1131 (88.8)	467 (92.5)	0.37	**
Income (in GBP/month), *mean* (*SD*)	*473.9* (*344.5*)	*486.4* (*324.6*)	*440.9* (*390.9*)	0.31	**
**Health behaviors**					
Smoking (past or current)	1175 (66.3)	833 (65.6)	342 (68.0)	1.00	0.34
Alcohol consumption (5+ days/week)	290 (20.0)	224 (20.4)	66 (18.9)	1.00	0.52
Physical inactivity^a^	1346 (75.7)	932 (73.2)	414 (82.0)	0.06	*
**Disability**					
Eyesight problem	312 (17.5)	199 (15.6)	113 (22.4)	0.46	*
Hearing problem	526 (29.6)	366 (28.7)	160 (31.7)	0.55	0.22
ADL difficulties (≥1)	442 (24.9)	280 (22.0)	162 (32.1)	0.05	*
IADL difficulties (≥1)	533 (30.0)	318 (25.0)	215 (42.6)	*	*
Mobility difficulties	618 (34.8)	473 (37.2)	145 (28.8)	0.26	*
**Physical health**					
Self‐rated health (fair/poor)	591 (34.5)	411 (32.5)	180 (40.3)	0.25	*
Long‐standing illness (limiting)	791 (44.5)	526 (41.3)	265 (52.5)	0.10	*
Diabetes	255 (14.3)	189 (14.8)	66 (13.1)	0.44	0.33
Hypertension	751 (42.2)	546 (42.8)	205 (40.6)	0.58	0.37
Cardiovascular disease	406 (22.8)	294 (23.1)	112 (22.2)	1.00	0.67
Lung disease/asthma	248 (13.9)	181 (14.2)	67 (13.3)	1.00	0.60
Arthritis	803 (45.2)	596 (46.8)	207 (41.0)	0.27	**
Osteoporosis	192 (10.8)	144 (11.3)	48 (9.5)	1.00	0.26
Cancer/blood disorder	95 (5.3)	68 (5.3)	27 (5.3)	1.00	1.00
Eye cataract	480 (27.0)	350 (27.5)	130 (25.7)	1.00	0.45
**Mental health**					
Dementia/Alzheimer's disease	132 (7.4)	72 (5.7)	60 (11.9)	0.27	*
Psychiatric condition/depressive symptoms^b^	370 (21.8)	256 (20.4)	114 (25.8)	0.19	**
**Cognition at recruitment**					
Self‐rated memory (fair/poor)	805 (47.3)	582 (46.3)	223 (50.3)	0.59	0.14
Orientation in time, *mean* (*SD*)	*3.3* (*1.3*)	*3.5* (*0.9*)	*2.7* (*1.8*)	*	*
Word‐list immediate recall, *mean* (*SD*)	*4.8* (*2.1*)	*5.0* (*2.0*)	*4.1* (*2.1*)	*	*
Word‐list delayed recall, *mean* (*SD*)	*3.1* (*2.4*)	*3.3* (*2.5*)	*2.4* (*2.3*)	**	*
Number series score^c^, *mean* (*SD*)	*517.4* (*35.6*)	*519.7* (*33.7*)	*507* (*41.8*)	0.28	*
Backward counting (correct)	1556 (91.7)	1177 (93.9)	379 (85.7)	**	*
Animal naming (number reported), *mean* (*SD*)	*17.8* (*7.8*)	*18.7* (*7.7*)	*15.5* (*7.8*)	*	*
Serial 7′s test, *mean* (*SD*)	*3.4* (*1.7*)	*3.6* (*1.6*)	*3.0* (*1.9*)	*	*
Object naming, *mean* (*SD*)	*4.4* (*1.0)*	*4.5* (*0.9*)	*4.2* (*1.2*)	*	*
Cognitive score (mTICS27), *mean* (*SD*)	*13.2* (*5.4*)	*13.9* (*5.2*)	*11.4* (*5.5*)	*	*
**Cognitive level (mTICS27 score)**					
Low (0–6)	277 (15.6)	118 (9.3)	159 (31.5)	*	*
Moderate (7–11)	491 (27.6)	353 (27.7)	138 (27.3)	1.00	–
Normal (12–27)	1010 (56.8)	802 (63.0)	208 (41.2)	*	–

*Notes*: Results are presented in *n*(*column%*) unless indicated otherwise. Missing data vary between variables. Significance level: *p‐value < 0.01, **p‐value < 0.05.

Abbreviations: ADL, (basic) activities of daily living; ELSA, English Longitudinal Study of Ageing; HCAP, Healthy Cognitive Ageing Project; IADL, instrumental activities of daily living; mTICS27, 27‐item modified Telephone Interview Cognitive Status; SD, standard deviation.

^a^ Hardly ever or never engaging in moderate or vigorous activities.

^b^ Depressive symptoms based on a 4+ cutoff on the CES‐D score.

^c^ Only administered in wave 8, nurse interview (invited *N* = 734, interviewed *N* = 599, not interviewed *N* = 135).

### 3.2 Participants versus non‐participants in ELSA‐HCAP

As per the above, the interviewed sample (*N* = 1273) resembles the invited sample (*N* = 1778) with respect to most baseline characteristics except for ADL/IADL and cognitive performance. A further comparison between the interviewed (*n* = 1273) and non‐participants (*n* = 505) within the invited sample highlighted specific characteristics associated with non‐participation at the bivariate level. Non‐participants were more likely to be older, not married (including widowed), and of lower education and income levels compared to participants (Table 2). Also, they were more likely to report poorer self‐rated health, a limiting long‐standing illness, arthritis, dementia or AD, and depressive symptoms compared to participants. Furthermore, they were more likely to have problems with hearing, difficulties with more than one ADL/IADL, and physical inactivity compared to participants. Finally, non‐participants were more likely to perform poorly on all cognitive tests (lower average mean scores, Table 2), with close to a 22% difference in the proportion of participants versus non‐participants in the mTICS27 low‐cognition category.

### 3.3 Factors associated with non‐participation in ELSA‐HCAP

In the base model, higher age (90+ years) was associated with almost four times the odds of non‐participation (OR 3.9 [95% CI 2.2–6.8]) compared to the reference age group of 60–69 years (Table 3). Additionally, those with any level of educational qualifications were 30% more likely to participate in the study compared to those with no qualifications. Other variables including gender, marital status, and income, were not significantly associated with non‐participation.

**TABLE 3:** Baseline adjusted models of factors associated with non‐participation in ELSA‐HCAP.

				Main analysis		Sensitivity analysis	
				Base model	Adjusted models^a^	Base model	Adjusted models^b^
Outcome: Non‐participation (vs. participation) in ELSA‐HCAP				*N* = 1778	*N* = 1778	*N* = 1684	*N* = 1684
Category	Variable	Reference group	Analysis group	OR (95% CI)	OR (95% CI)	OR (95% CI)	OR (95% CI)
**Sociodemographics and economic measures**	Age in years	60–69	70–79	1.28 (0.96–1.69)		1.16 (0.87–1.55)	
			80–89	1.28 (0.93–1.75)		1.02 (0.73–1.44)	
			90+	**3.90 (2.24**–**6.77)**	^c^	**2.50 (1.35**–**4.63)**	^c^
	Gender	Men	Women	0.99 (0.79–1.23)		1.09 (0.86–1.39)	
	Marital status	Married	Not married	1.00 (0.76–1.30)		0.93 (0.70–1.23)	
	Education	No qualifications	Any qualifications	**0.68 (0.54**–**0.86)**	^c^	**0.71 (0.55**–**0.90)**	^c^
	Income in GBP/month			1.00 (1.00–1.00)		1.00 (1.00–1.00)	
**Behavioral and physical health measures**	Physical activity	Active	Inactive^d^		**1.32 (1.01**–**1.75)**		1.20 (0.90–1.59)
	Self‐rated health	Good/very good	Fair/poor		**1.27 (1.00**–**1.60)**		1.07 (0.83–1.38)
	Long‐standing illness	None/not limiting	Limiting		**1.34 (1.07**–**1.67)**		1.13 (0.89–1.43)
	Arthritis	Not reported	Reported		**0.77 (0.61**–**0.96)**		**0.76 (0.60**–**0.96)**
**Disability measures**	Eyesight	Very good/good	Fair/poor		1.30 (0.99–1.71)		1.20 (0.89–1.62)
	Hearing	Very good/good	Fair/poor		0.93 (0.73–1.18)		0.86 (0.66–1.12)
	ADL difficulties	None reported	≥1		**1.31 (1.02**–**1.67)**		1.16 (0.88–1.52)
	IADL difficulties	None reported	≥1		**1.78 (1.40**–**2.26)**		**1.56 (1.20**–**2.02)**
	Mobility difficulties	Not reported	Reported		0.84 (0.66–1.07)		0.93 (0.73–1.20)
**Mental health measures**	Dementia/Alzheimer's disease	Not reported	Reported		**1.55 (1.02**–**2.33)**		1.30 (0.82–2.07)
	Psychiatric condition/depressive symptoms^e^	Absent	Present		**1.34 (1.03**–**1.75)**		1.22 (0.91–1.62)
**Cognition measure**	mTICS27 cognitive score^f^				**0.93 (0.91**–**0.95)**		**0.94 (0.91**–**0.96)**

*Note*: In bold are estimates with *p*‐value < 0.05.

Abbreviations: ADL, (basic) activities of daily living; CI, confidence interval; ELSA, English Longitudinal Study of Ageing; HCAP, Healthy Cognitive Ageing Project; IADL, instrumental activities of daily living; mTICS27, 27‐item modified Telephone Interview Cognitive Status; OR, odds ratio.

^a^ Separate models were conducted for each measure adjusting for age, gender, marital status, education, and income in the total invited sample *N* = 1778.

^b^ Separate models were conducted for each measure adjusting for age, gender, marital status, education, and income in the total eligible sample *N* = 1684.

^c^ Age and education remained significant in all the separate models except for the model with the cognition measure, where only age was significant.

^d^ Hardly ever or never engaging in moderate or vigorous activities.

^e^ Depressive symptoms based on a 4+ cutoff on the CES‐D score.

^f^ A higher score on the mTICS27 indicates better cognition.

In the demographic‐adjusted models, physical inactivity, having a limiting long‐standing illness, poor self‐rated health, and difficulties in performing ADL and IADL were each associated with higher adjusted odds of non‐participation (Table 3). Similarly, having difficulties in ADL and IADL, a self‐reported diagnosis of dementia/AD, and depression were associated with higher adjusted odds of non‐participation. A higher cognition score (indicative of a better cognitive performance) was associated with participation (OR 0.93, [0.91–0.95]). Finally, having arthritis and hearing problems were associated with a greater likelihood of taking part. These factors were commonly reported in the invited sample (45% arthritis and 30% hearing problems), as shown in Table 2.

**FIGURE 2:**
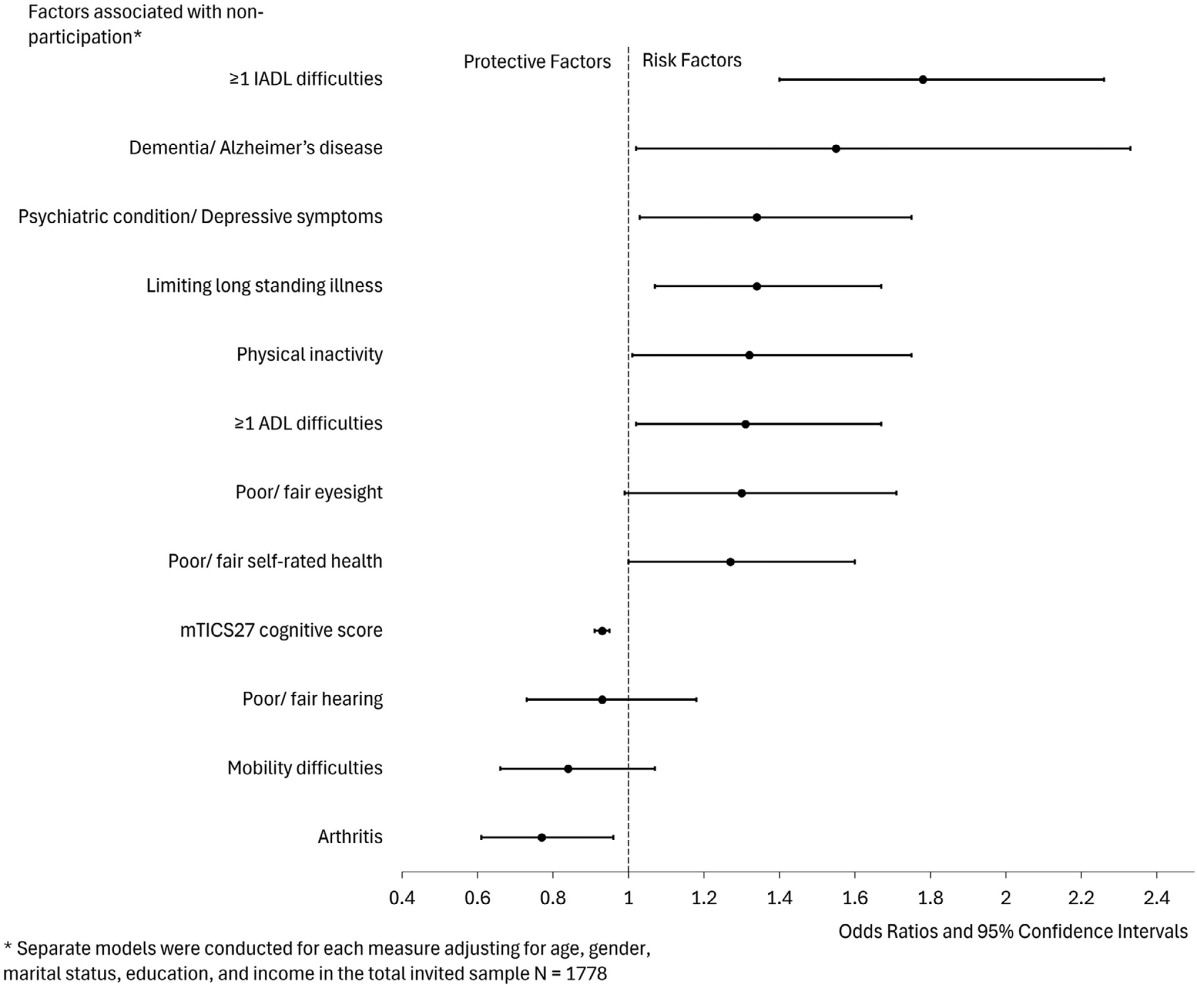
Health and behavioral factors associated with non‐participation in ELSA‐HCAP (*N* = 1778). This figure shows the odds ratios and their 95% CI for the associations between health and behavioral factors with non‐participation (outcome). Those with and OR below 1 are labeled protective factors while those with and OR above 1 are labeled as risk factors. The main factors contributing to non‐participation are having at least one difficulty with activities of daily living and having a low‐cognition score (mTICS27 scale). ADL, (basic) activities of daily living; CI, confidence interval; ELSA, English Longitudinal Study of Ageing; HCAP, Healthy Cognitive Ageing Project; IADL, Instrumental activities of daily living; mTICS27, the modified Telephone Interview Cognitive Status (27 items); OR, odds ratio.

These results are summarized in Figure 2 in increasing order of OR, showing that the main factors associated with non‐participation are IADL difficulties, dementia/AD, and depression. In all the demographic‐adjusted models, higher age and low education remained significant factors associated with non‐participation, except with cognition, where only higher age was significant. The results of the sensitivity analysis, excluding the non‐reachable cases (91 died and three moved out of Britain), showed that higher age, IADL difficulties, and low cognition remained significant risk factors for non‐participation (Table 3).

### 3.4 Participants’ baseline characteristics using weighted and unweighted data

Differences in means and proportions were observed when comparing weighted and unweighted data for the participants’ baseline characteristics. These were most evident for age, working status, self‐reported dementia diagnosis, self‐rated memory, and cognition measures at recruitment, where significantly lower means and proportions were observed in the weighted data compared to the unweighted one (Table 4). Also, lower proportions were observed for poor physical health measures, including long‐term conditions, eyesight and hearing problems, and IADL difficulties, but with no significant differences in means or proportions between the two samples. Finally, few or no differences were observed for other sociodemographic, economic, and health behavior measures.

**TABLE 4:** Baseline characteristics of the interviewed ELSA‐HCAP sample using unweighted and weighted data.

	Unweighted *N* = 1273		Weighted *N* = 1273		
Characteristics	Mean	%	Mean	%	*p*‐Value of z‐test (difference in means/proportions)
**Sociodemographics**					
Age (in years)	74.7		73.3		*
Gender (women)		55.0		54.0	0.62
Marital status (not married)		36.4		33.9	0.18
Ethnicity (White)		96.7		97.1	0.58
Education (no qualifications)		33.1		31.2	0.29
Working status (not in work)		88.8		84.9	*
Income (in GBP/month)	486.4		510.9		0.06
**Health behaviors**					
Smoking (past or current)		65.6		65.6	0.98
Alcohol consumption (5+ days/week)		20.4		21.5	0.52
Physical inactivity^a^		73.2		72.2	0.56
**Disability**					
Eyesight problem		15.6		13.9	0.22
Hearing problem		28.7		26.3	0.16
ADL difficulties (≥1)		22.0		22.2	0.91
IADL difficulties (≥1)		25.0		22.9	0.22
Mobility difficulties		37.2		38.6	0.45
**Physical health**					
Self‐rated health (fair/poor)		32.5		29.7	0.12
Long‐standing illness (limiting)		41.3		40.7	0.75
Diabetes		14.8		13.9	0.50
Hypertension		42.8		41.1	0.36
Cardiovascular disease		23.1		20.7	0.14
Lung disease/asthma		14.2		13.4	0.57
Arthritis		46.8		46.0	0.68
Osteoporosis		11.3		10.4	0.48
Cancer/blood disorder		5.3		4.8	0.59
Eye cataract		27.5		25.5	0.27
**Mental health**					
Dementia/Alzheimer's disease		5.7		2.6	*
Psychiatric condition/depressive symptoms^b^		20.4		20.3	0.95
**Cognition at recruitment**					
Self‐rated memory (fair/poor)		46.3		40.0	*
Orientation in time	3.5		3.6		0.02**
Word‐list immediate recall	5.0		5.7		*
Word‐list delayed recall	3.3		4.1		*
Number series score^c^	519.7		523.4		0.05
Backward counting (correct)		93.9		96.5	*
Animal naming (number reported)	18.7		20.2		*
Serial 7′s test	3.6		3.9		*
Object naming	4.5		4.6		*
Cognitive score (mTICS27)	13.9		15.7		*
**Cognitive level (mTICS27 score)**					
Low (0–6)		9.3		6.1	*
Moderate (7–11)		27.7		13.0	*
Normal (12–27)		63.0		80.9	*

*Notes*: Results are presented in *n*(*column%*) unless indicated otherwise. Missing data vary between variables. Significance level: *p‐value < 0.01, **p‐value < 0.05.

Abbreviations: ADL, (basic) activities of daily living; ELSA, English Longitudinal Study of Ageing; HCAP, Healthy Cognitive Ageing Project; IADL, instrumental activities of daily living; mTICS27, 27‐item modified Telephone Interview Cognitive Status; SD, standard deviation.

^a^ Hardly ever or never engaging in moderate or vigorous activities.

^b^ Depressive symptoms based on a 4+ cutoff on the CES‐D score.

^c^ Only administered in wave 8, nurse interview (invited *N* = 734, interviewed *N* = 599, not interviewed *N* = 135).

## 4 DISCUSSION

In nationally sampled studies, it is vital to understand who takes up invitations to participate and what might influence loss‐to‐follow‐up or invitation to take part in sub‐studies to make accurate inferences. Compared with other longitudinal studies of older people in the UK and mainland Europe, ELSA‐HCAP shows similar initial response and attrition rates.^9^, ^10^ Therefore, understanding the ELSA‐HCAP sample is important to identify any systematic differences between those who took part in the study and those who did not, which may affect the generalizability of study findings. Statistical analysis of the invited sample, including participants and non‐participants, helps examine the extent of the participation bias by testing the association of study variables with the participation outcome.^6^, ^19^

In our study, we showed that the participation bias occurred in ELSA‐HCAP despite the oversampling from those in ELSA with low cognition at recruitment and the observed similarities in baseline characteristics between the interviewed and invited samples. In line with similar analyses from other population‐based aging studies, sociodemographic and health factors associated with non‐participation in our sample include the older age group, lower education, lower income, and higher prevalence of long‐term conditions.^20^, ^21^ The comparison of the profiles between participants and non‐participants also highlights the importance of adjusting models for potential risk factors to non‐participation, in particular, physical inactivity, eyesight problems, ADL/IADL disability, and long‐standing illness. Other factors like diabetes, hypertension, and osteoporosis were not strongly associated with non‐participation at the bivariate level, which can be explained by the protective effect of medication,^22^ the associated survival bias,^9^ and/or the small number of cases reporting these conditions in the invited sample.^23^ The multivariable analysis of the factors associated with non‐participation further showed the independent associations of 90+ age group, IADL difficulties, and lower cognition with non‐participation. The protective effect of arthritis and hearing problems is likely due to survival bias associated with more participants reporting these conditions in older age.^24^, ^25^ Adjusting for these measures when analyzing data from the ELSA‐HCAP study is essential to correct for this participation bias.

Sample weights have been included in the ELSA‐HCAP dataset to adjust estimates for non‐response and maintain the representativeness of those 65 years and older in ELSA. A comparison of weighted to unweighted data showed that the sample representation of the distribution of disability measures, physical and mental health measures, and cognition scores remains biased. For example, there was a 3.2% difference in the proportion of those with low cognition in the weighted versus unweighted sample, reflecting an overestimation of the low cognition group. Consequently, the use of weights in the analysis of ELSA‐HCAP data is essential to make correct inferences on cognition in the 65+‐year‐old English population and to make robust comparisons of cognitive function across countries, a primary aim of the HCAP sister studies.^26^

Among the factors associated with non‐participation, age, cognition, working status, long‐standing illness, physical inactivity, and self‐rated health were part of the model used to derive the weights.^16^ Nevertheless, the comparison of weighted to unweighted samples did not show differences for these factors, except for cognition measures, working status, and age. Other factors, such as ADL difficulties, eyesight problems, previous dementia/AD diagnosis, and depression, were not included in the weights’ derivation model. Hence, we can argue that adjusting for these factors in weighted models can be useful in further correcting for the non‐participation bias.

This type of selection bias is a common limitation of many population‐based studies where the outcome of interest (e.g., cognition) is independently related to non‐response.^27^ Additional efforts to encourage and facilitate participation of those with low cognition are required. Studies can examine reasons for refusal or non‐response and consider alternative or more targeted sampling strategies to recruit those from the poor cognition groups.

In conclusion, cohort studies, a vital source for estimating the prevalence and incidence of dementia, could benefit from more accurate and transparent reporting of non‐participation bias and advice on the use and limitations of statistical solutions for correct inferences. This study highlighted the factors associated with participation bias in the ELSA‐HCAP data and the use of sample weights along with adjusting for these factors in the modeling of the data with the aim of providing a better understanding of the study sample and how to use this rich resource to capture the spectrum of cognitive change in older age within an internationally comparative framework.

## CONFLICT OF INTEREST STATEMENT

The authors declare no conflicts of interest. Author disclosures are available in the Supporting Information.

## CONSENT STATEMENT

The ELSA‐HCAP study was conducted in accordance with the Declaration of Helsinki, and ethical approval and experimental protocols were granted by the South Central—Berkshire Research Ethics Committee (REC Ref: 17/SC/0254). All participants gave their informed consent to take part in the study.

## Supporting information



Supporting Information

Supporting Information
