# Supplementary material for: Factors associated with non‐participation in the Healthy Cognitive Ageing Project
Source: Alzheimers Dement. 2025 Apr 28;21(4):e70169. doi: 10.1002/alz.70169 (PMC12034936; doi:10.1002/alz.70169)
Supplement: Supplementary file 1 — Supporting Information [file ALZ-21-e70169-s002.docx]

Supplementary material

## Supplementary Table 1: A summary of the cognition tests assessed in ELSA waves 7 and 8.

continued

| Domain | Test* | Description | Scoring | References† |
| --- | --- | --- | --- | --- |
| Memory | Orientation in time | Participants are asked to state the current date, month, year, and day of the week. | Scored based on the number of correct responses (score ranges between 0 to 4). | Commonly used in cognitive assessments (2) |
|  | Immediate and delayed word-list recall | Participants are read a list of 10 words and asked to recall as many as possible immediately and after a delay. | Scored based on the number of words correctly recalled immediately and after the delay (each score ranges between 0 to 10). | Commonly used in cognitive assessments (3) |
| Fluid intelligence | Number series score | Participants are given a series of 6 number sequences in increasing order of difficulty and asked to identify the missing number(s) in the sequence. | Scored based on the HRS 2012 code. | Health and Retirement Study (HRS) derivation SAS code (4)  Stata code for derivation in ELSA (5) |
| Executive function  Executive function (continued) | Backwards counting | Participants are asked to count backwards from 20 to when stopped by the interviewer with one chance to start again. | Scored 1 if correct based on counting backwards from 19 to 10 or from 20 to 11 without error in the first or second trial, and 0 if incorrect/ don’t know.  When used as part of the 27-item modified Telephone Interview Cognitive Status (mTICS27) score, it is scored as 2 if correct in first trial, 1 if correct in second trial, and 0 if incorrect/ don’t know. | Commonly used in cognitive assessments (6) |
|  | Animal naming | Participants are asked to name as many animals as possible within one minute. This is a measure of verbal fluency. | Scored based on the total number of unique animals named (range 0 to 100) | Commonly used in cognitive assessments (7) |
|  | Serial 7’s subtractions | Participants are asked to subtract 7 from 100 and continue doing so up to 5 times. | Scored based on the number of correct subtractions (score ranges between 0 to 5). | Part of the Mini-Mental State Examination (MMSE) (8, 9) |
|  | Object naming | Participants are asked to name objects (scissors, cactus) and people (Monarch, Prime minister, and US President). | Scored based on the number of correct answers (score ranges between 0 to 5). | Has similar properties to the Boston Naming Test (10) |

*All the cognitive tests have equivalent tests in the Health and Retirement Study (HRS). (1)

†Please refer to the reference list below.

**Reference list for Supplementary Table 1:**

1. Sonnega A, Faul JD, Ofstedal MB, Langa KM, Phillips JW, Weir DR. Cohort Profile: the Health and Retirement Study (HRS). Int J Epidemiol. 2014 Apr;43(2):576-85. doi: 10.1093/ije/dyu067.
2. O’Keeffe E, Mukhtar O, O’Keeffe ST. Orientation to time as a guide to the presence and severity of cognitive impairment in older hospital patients. J Neurol Neurosurg Psychiatry. 2010;82(5):500. doi: 10.1136/jnnp.2010.214817.
3. Fayemiwo MA, Olowookere TA, Olaniyan OO, Ojewumi TO, Oyetade IS, Freeman S, Jackson P. Immediate word recall in cognitive assessment can predict dementia using machine learning techniques. Alzheimers Res Ther. 2023;15:111. doi: 10.1186/s13195-023-01250-5.
4. Ofstedal MB, Fisher GG, Herzog AR. Documentation of Cognitive Functioning Measures in the Health and Retirement Study. HRS/AHEAD Documentation Report. Ann Arbor, MI: University of Michigan; 2005. Available from: <https://hrs.isr.umich.edu/sites/default/files/biblio/dr-027b.pdf>
5. Assaad S. ELSA-HCAP study: Derivation of the summary variables for the Number Series Test. STATA 17 code, version 1.0. 2023. Available from: <https://github.com/SarahAssaad/ELSA_HCAP_Number_Series>
6. Smith A. The Serial Sevens Subtraction Test. Arch Neurol. 1967;17(1):78-80. doi: 10.1001/archneur.1967.00470250082008.
7. Campagna F, Montagnese S, Ridola L, Senzolo M, Schiff S, De Rui M, et al. The animal naming test: An easy tool for the assessment of hepatic encephalopathy. Hepatology. 2017;66(1):198-208. doi: 10.1002/hep.29146.
8. Smith A. The Serial Sevens Subtraction Test. Arch Neurol. 1967;17(1):78-80. doi: 10.1001/archneur.1967.00470250082008.
9. Folstein MF, Folstein SE, McHugh PR. “Mini-mental state”. A practical method for grading the cognitive state of patients for the clinician. J Psychiatr Res. 1975 Nov;12(3):189-98. doi: 10.1016/0022-3956(75)90026-6.
10. Kaplan E, Goodglass H, Weintraub S. The Boston Naming Test. Philadelphia: Lea & Febiger; 1983.

Supplementary Information 1: Derivation of the mTICS27 score for ELSA-HCAP

The 27-item modified Telephone Interview Cognitive Status (mTICS27) scale consists of the following cognitive tests: immediate word-list recall (range 0 to 10), delayed word-list recall (range 0 to 10), serial 7’s subtractions (range 0 to 5), and the backward counting (range 0 to 2). For each one of these contributing scores, a ‘don’t know’ answer was coded as zero while a ‘refused’ or ‘not applicable’ answer was coded as missing. Scores can range from 0 to 27, with higher scores indicating better performance. The invited sample was then classified into one of three cognitive groups based on their valid mTICS27 score: low (mTICS27 <= 6), moderate (mTICS27 7 to 11), and normal (mTICS27 >=12). These cognition groups are different from the ones used for the participants’ selection in ELSA-HCAP,^[[1]](#footnote-1)^ which included a dementia/Alzheimer’s Disease diagnosis in the low cognition group. This explains the differences in the cognitive group-specific response rates in Table 1 as well as the means and proportion of the cognitive measures in this paper compared to the cohort profile paper.^1^

The mTICS27 score was assigned a missing value if at least one of the contributing tests had a missing value. A total of 92 out of the 1778 invited participants had missing data on the mTICS27. These were scrutinised by reviewing the scores on the tests they had completed as part of the mTICS27, as well as performance on other cognitive tests administered in ELSA at time of recruitment (waves 7 or 8). Three cases were classified as having ‘normal cognition’ when the incomplete mTICS27 score>=12 and there was no previous self-report of dementia/Alzheimer’s Disease (AD) diagnosis in ELSA. An additional 2 cases with incomplete mTICS27 score>=7, high scores on other cognitive tests, and no report of dementia/AD diagnosis were also classified as having ‘normal cognition’. The remaining cases (n= 87) had a previous self-reported dementia/AD diagnosis in ELSA and zero/missing/not applicable score on 3 or more cognitive tests and were, thus, classified as having ‘low cognition’.

Supplementary Information 2: Characteristics of HCAP eligible versus interviewed samples

Recruitment to ELSA-HCAP was designed to oversample people with poor cognitive function.^[[2]](#footnote-2)^ Selection was based on cognitive performance measured using the modified Telephone Interview Cognitive Status (mTICS) in waves 8 or 7 and/or having previously reported a dementia diagnosis at any point in waves 1-8.^1,^^[[3]](#footnote-3)^ Three groups were defined using this information, and participants were categorised into low, moderate, and normal ‘cognition groups’. To maximise on those with poor cognition, all the eligible participants for the lowest cognition group were included in the invited sample.

Because of the sample derivation method of oversampling of the poor cognition group, higher proportion of participants were expected to have low cognition. The results of the supplementary analysis showed that 9.3% and 27.7% of participants were in the low and moderate cognitive groups in the interviewed sample compared to 5.1% and 11.7% in the eligible sample, respectively (Supplementary Table S2). The performance of participants on the cognitive tests was significantly lower than those in the eligible sample (except for ‘orientation in time’). The average age was higher for participants (74.7 vs 73.8) compared with those eligible to participate. Those interviewed had a higher proportion with lower education and lower total net income compared to eligible samples. Also, the interviewed group had higher proportions of poor self-rated health, hypertension, CVD, arthritis, osteoporosis, dementia or AD, hearing problems, mobility difficulties, and physical inactivity compared to eligible individuals.

Supplementary Table 2: Differences in profiles between eligible and interviewed samples in the ELSA-HCAP study.

| **Variables (described group)** | **HCAP Eligible**  **N = 5715** | **Interviewed**  **N = 1273** | **p-value** |
| --- | --- | --- | --- |
| **Socio-demographics** |  |  |  |
| Age (in years), *Mean (SD)* | *73.8 (7.6)* | *74.7 (7.5)* | * |
| Gender (women) | 3224 (56.4) | 700 (55.0) | 0.52 |
| Marital status (not married) | 2005 (35.1) | 463 (36.4) | 0.50 |
| Ethnicity (white) | 5562 (97.3) | 1231 (96.7) | 1.00 |
| Education (no qualifications) | 1521 (27.0) | 416 (33.1) | * |
| Income (in GBP per month), *Mean (SD)* | *530.3 (429.1)* | *486.4 (324.6)* | * |
| Working status (not in work) | 4999 (87.5) | 1131 (88.8) | 0.05 |
| **Health behaviours** |  |  |  |
| Smoking (past or current) | 3703 (65.1) | 833 (65.6) | 0.50 |
| Alcohol consumption (5+ days/week) | 1018 (20.6) | 224 (20.4) | 0.46 |
| Physical inactivity^‡^ | 3939 (68.9) | 932 (73.2) | * |
| **Disability** |  |  |  |
| Eyesight problem | 857 (15.0) | 199 (15.6) | 0.37 |
| Hearing problem | 1461 (25.6) | 366 (28.7) | † |
| ADL difficulties (≥1) | 1130 (19.8) | 280 (22.0) | 0.11 |
| IADL difficulties (≥1) | 1325 (23.2) | 318 (25.0) | 0.13 |
| Mobility difficulties | 2264 (39.6) | 473 (37.2) | † |
| **Physical Health** |  |  |  |
| Self-rated health (fair/poor) | 1624 (29.4) | 411 (32.5) | † |
| Long standing illness (limiting) | 2273 (39.8) | 526 (41.3) | 0.51 |
| Diabetes | 720 (12.6) | 189 (14.8) | 0.06 |
| Hypertension | 2303 (40.3) | 546 (42.8) | † |
| Cardiovascular disease | 1170 (20.5) | 294 (23.1) | † |
| Lung disease/ Asthma | 775 (13.6) | 181 (14.2) | 1.00 |
| Arthritis | 2457 (43.0) | 596 (46.8) | * |
| Osteoporosis | 522 (9.1) | 144 (11.3) | † |
| Cancer/ Blood disorder | 286 (5.0) | 68 (5.3) | 1.00 |
| Eye cataract | 1447 (25.3) | 350 (27.5) | 0.14 |
| **Mental Health** |  |  |  |
| Dementia/ Alzheimer’s Disease | 136 (2.4) | 72 (5.7) | * |
| Psychiatric condition/ Depressive symptoms^§^ | 1054 (19.2) | 256 (20.4) | 0.42 |
| **Cognition at recruitment** |  |  |  |
| Self-rated memory (fair/ poor) | 2157 (39.2) | 582 (46.3) | * |
| Orientation in time, *Mean (SD)* | *3.5 (1.1)* | *3.5 (0.9)* | 1.00 |
| Word-list immediate recall, *Mean (SD)* | *5.7 (1.8)* | *5.0 (2.0)* | * |
| Word-list delayed recall, *Mean (SD)* | *4.3 (2.3)* | *3.3 (2.5)* | * |
| Number series score^¶^, *Mean (SD)* | *528.2 (30.4)* | *519.7 (33.7)* | * |
| Backwards counting (correct) | 5269 (96.22) | 1177 (93.9) | * |
| Animal naming (number reported), *Mean (SD)* | *20.4 (7.4)* | *18.7 (7.7)* | * |
| Serial 7’s test, *Mean (SD)* | *4.1 (1.4)* | *3.6 (1.6)* | * |
| Object naming, *Mean (SD)* | *4.7 (1.0)* | *4.5 (0.9)* | * |
| Cognitive score (mTICS), *Mean (SD)* | *16.1 (4.6)* | *13.9 (5.2)* | * |
| Cognitive level (mTICS27 score) |  |  |  |
| Low (0-6) | 282 (5.1) | 118 (9.3) | * |
| Moderate (7-11) | 645 (11.7) | 353 (27.7) | * |
| Normal (12-27) | 4599 (83.2) | 802 (63.0) | * |

**Notes:** Results are presented in *n(column%)* unless indicated otherwise. Missing data varies between variables. Significance level: *p-value <0.01, †p-value <0.05. ^‡^ Hardly ever or never engaging in moderate or vigorous activities. ^§^Depressive symptoms based on a 4+ cut-off on the CES-D score. ^¶^ Only administered in wave 8, nurse interview (eligible N =2446, interviewed N= 599).

**Abbreviations**: ADL, activities of daily living; IADL, instrumental activities of daily living; mTICS27, 27-item modified Telephone. Interview Cognitive Status; SD, standard deviation.

1. Cadar D, Abell J, Matthews FE, Brayne C, Batty GD, Llewellyn DJ, Steptoe A. Cohort Profile Update: The Harmonised Cognitive Assessment Protocol Sub-study of the English Longitudinal Study of Ageing (ELSA-HCAP). Int J Epidemiol. 2021;50(3):725-6i. [↑](#footnote-ref-1)
2. 1 Cadar D, Abell J, Matthews FE, Brayne C, Batty GD, Llewellyn DJ, Steptoe A. Cohort Profile Update: The Harmonised Cognitive Assessment Protocol Sub-study of the English Longitudinal Study of Ageing (ELSA-HCAP). Int J Epidemiol. 2021;50(3):725-6i. [↑](#footnote-ref-2)
3. 2 Brandt J, Spencer M, Folstein M. The telephone interview for cognitive status. Neuropsychiatry Neuropsychol Behav Neurol. 1988;1(2):111-7. [↑](#footnote-ref-3)
